# Transthyretin Amyloid Fibril Disrupting Activities of Extracts and Fractions from *Juglans mandshurica* Maxim. var. *cordiformis* (Makino) Kitam.

**DOI:** 10.3390/molecules24030500

**Published:** 2019-01-30

**Authors:** Niraj Chaudhary, Ryoko Sasaki, Tsuyoshi Shuto, Masato Watanabe, Teppei Kawahara, Mary Ann Suico, Takeshi Yokoyama, Mineyuki Mizuguchi, Hirofumi Kai, Hari Prasad Devkota

**Affiliations:** 1 Department of Molecular Medicine, Graduate School of Pharmaceutical Sciences, Kumamoto University, 5-1 Oe-honmachi, Chuo-ku, Kumamoto 862-0973, Japan; nirajchaudharypu@gmail.com (N.C.); 188y1008@st.kumamoto-u.ac.jp (R.S.); tshuto@gpo.kumamoto-u.ac.jp (T.S.); mann@gpo.kumamoto-u.ac.jp (M.A.S.); 2 Program for Leading Graduate Schools, Health Life Science: Interdisciplinary and Glocal Oriented (HIGO) Program, Kumamoto University, Kumamoto 862-0973, Japan; 3 Department of Medicinal Botany, Graduate School of Pharmaceutical Sciences, Kumamoto University, Kumamoto 862-0973, Japan; wata-m@gpo.kumamoto-u.ac.jp; 4 Useful and Unique Natural Products for Drug Discovery and Development (UpRoD), Program for Building Regional Innovation Ecosystems at Kumamoto University, Kumamoto 862-0973, Japan; teppei@kumamoto-u.ac.jp; 5 Graduate School of Medicine and Pharmaceutical Sciences, University of Toyama, 2630 Sugitani, Toyama 930-0194, Japan; tyokoya3@pha.u-toyama.ac.jp (T.Y.); mineyuki@pha.u-toyama.ac.jp (M.M.)

**Keywords:** transthyretin-related amyloidosis, transthyretin protein (TTR), *Juglans mandshurica* var. *sachalinensis*, *Juglans mandshurica* var. *cordiformis*, naphthoquinone, juglone

## Abstract

Transthyretin-related amyloidosis is a slowly progressive disorder caused by deposition of insoluble amyloid plaques formed by fibrillization of mutant or defective transthyretin (TTR) monomers that leads to neurodegeneration and organ failure. Thus, any compound exhibiting TTR amyloid formation inhibitory activity or TTR amyloid fibril disrupting activity might be a potential candidate for the development of therapies for these disorders. Our aim in this study was the evaluation of the TTR amyloid fibril disrupting potential of extracts of leaves and immature fruits of two *Juglans* plants, i.e., *Juglans mandshurica* var. *sachalinensis* and *Juglans mandshurica* var. *cordiformis*. The TTR amyloid fibril disrupting activity was measured by Thioflavin-T (ThT) assay and PROTEOSTAT^®^ Protein aggregation assay methods. A fifty percent acetone extract of the fruits of *Juglans mandshurica* var. *cordiformis* showed strong amyloid fibril disrupting activity, and was further fractionated using different solvents. Ethyl acetate and *n*-butanol fractions showed significant activity in both assays. Syringic acid was isolated and identified as main compound in both of these fractions; however, it did not show any activity. Furthermore, some of the previously reported compounds from *Juglans* plants including naphthoquinone derivatives and phenolic compounds were evaluated to identify the potential bioactive compounds. Among them, juglone, a naphthoquinone derivative showed promising activity. However, juglone also showed strong cytotoxicity in HEK293 cells. Thus, future studies should focus on the isolation and identification of naphthoquinone derivatives or other compounds from *Juglans* plan ts with potent bioactivity and low cytotoxicity.

## 1. Introduction

Transthyretin-related amyloidosis is a slowly progressive disease characterized by the abnormal deposition of amyloid fibrils in the body’s organs that lead to polyneuropathy or cardiomyopathy [1]. The amyloid (fibrils) deposits are the composite of accumulated TTR protein whose physiological function is to transport thyroxine (T4) hormone and retinol-binding protein (RBP) through the blood circulation. However, in TTR amyloidosis either due to single amino acid substitutions of TTR monomers or denaturation conditions, TTR tetramer disassembles into monomers following partial unfolding, and mis-assembles into amyloid fibril [2]. Amyloid fibrils formation of mutant and wild-type TTR are responsible for ATTRm and ATTRwt amyloidosis, respectively [3]. More than 100 different TTR variants are associated with ATTRm amyloidosis, of which the V30M mutation is the most common and prevalent in Japan, Portugal and Sweden [4]. The structural study of TTR fibril revealed that the oligomerization of monomeric filaments occurs very slowly under physiological conditions and implicates the conformational change from helical to β-pleated sheet conformation to form amorphous aggregates, which are resistant to degradation. There are only limited therapeutic strategies against TTR amyloidosis such as orthotopic liver transplantation [5], use of tetramer stabilizers (diflunisal, tafamidis) [6] and TTR silencers (inotersen) [7,8]. However, many patients continue to have disease progression or neurologic impairment following the treatments [9,10]. Consequently, several possibilities are being explored to slow or reverse fibrillary proteins’ aggregation pathway, including the β-sheet breaker (amyloid-disrupter) mechanism [11]. Thus, the exploration of amyloid fibril disrupting compounds derived from plant sources is also significant. Correspondingly, many of the naturally derived constituents possess various structural backbones and divergent biological functions like anti-oxidant, anti-inflammatory and metal chelating capacities that can be employed for rational drug screening strategy to find potent anti-amyloidosis agents [12,13].

*Juglans* plants (Family: Juglandaceae) are fast-growing deciduous trees widely distributed in eastern Asia [14,15]. Various parts of *Juglans* plants are used as traditional medicines in China, Korea and Japan for many illnesses like gastric related diseases, uterine prolapse and leukopenia chiefly [16]. Previous studies reported that *J. regia* can inhibit amyloid-β (Aβ) fibrillization and solubilize its fibrils [17]. Similarly, oral treatment with walnuts enhanced memory deficit and learning behavior in Alzheimer’s disease transgenic model mouse [18]. Immature fruits, mature fruit parts (kernels, shells and seeds), bark and leaves of *Juglans* mainly contain naphthoquinone derivatives, naphthalenyl glucosides, phenolic acids, flavonoids, tetralones, terpenoids, diarylheptanoids and galloyl glycosides [19]. Naphthoquinone derivatives and naphthalenyl glycosides showed significant anti-bacterial, -fungal, -viral and insecticidal properties [20]. They also have anti-fatigue, antioxidant and immunoregulatory effects [21]. Polyphenolic compounds including flavonoids, have potent antioxidant activity [22] and have been reported to reduce stroke and coronary heart disease risk factors [23]. 

**Figure 1:**
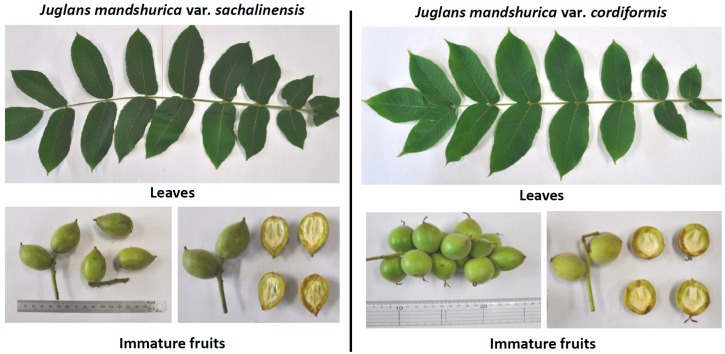
The photographs of leaves and immature fruits of *J. mandshurica* var. *sachalinensis* and *J. mandshurica* var. *cordiformis*.

During our preliminary screening of several plant extracts for potential amyloid fibril disrupting activity, the extracts of the leaves and immature fruits of *Juglans* plants showed potent activity. In this study, our main objectives were the evaluation of the TTR amyloid fibril disrupting activity of extracts of leaves and immature fruits of *Juglans mandshurica* Maxim. var. *sachalinensis* (Komatsu) Kitam. and *Juglans mandshurica* Maxim. var. *cordiformis* (Makino) Kitam. (Figure 1), along with previously reported chemical constituents from *Juglans* plants. 

## 2. Results

### 2.1. Screening of Extracts of Juglans Plants for TTR Amyloid Fibril Disrupting Activity

**Figure 2:**
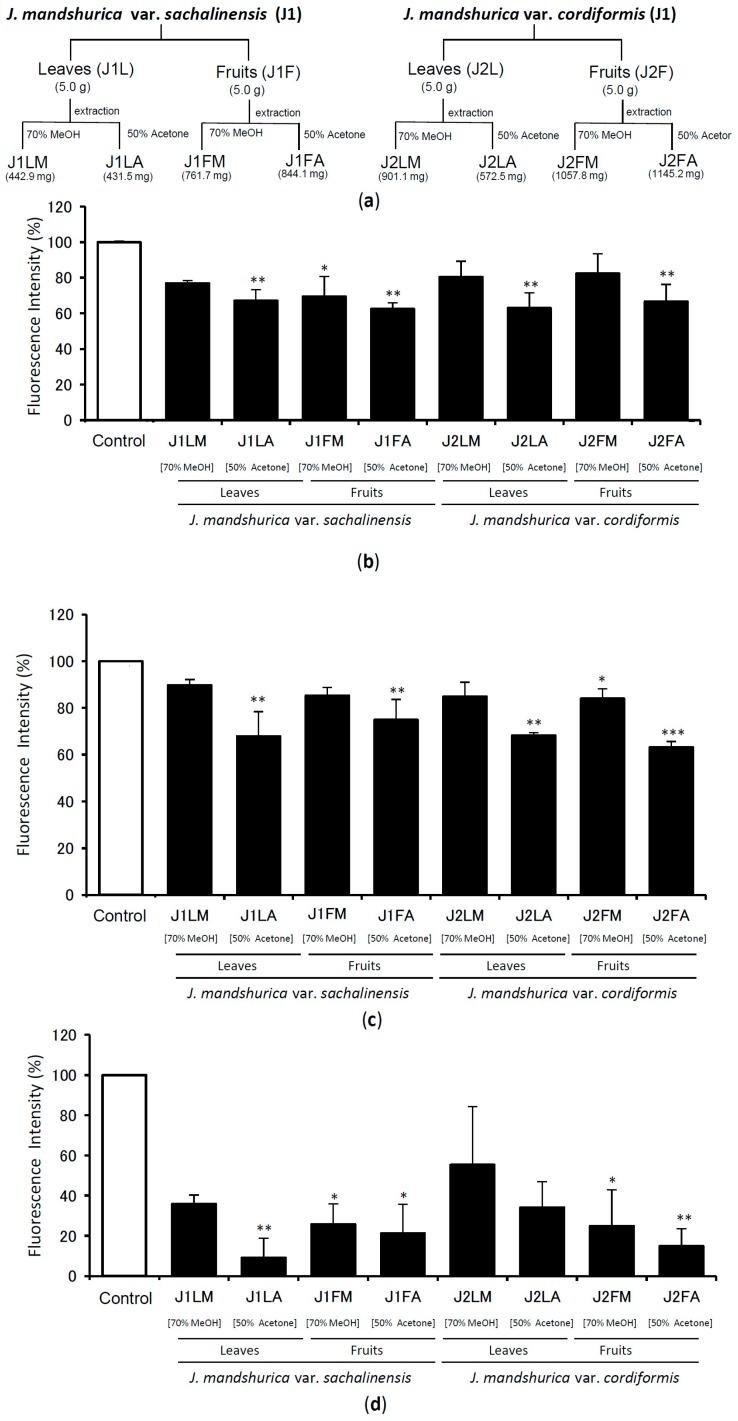
The extraction of leaves and immature fruits of *J. mandshurica* var. *sachalinensis* and *J. mandshurica* var. *cordiformis* with 70% methanol and 50% acetone at room temperature (**a**). The amyloid fibril disrupting activity of different extracts (0.5 mg/mL) when incubated with V30M-TTR tetramer (**b**) and WT-TTR tetramer (**c**) for 24 h, then quantified with ThT assay. Quantification was performed by with PROTEOSTAT^®^ Protein Aggregation Assay following similar procedure using V30M-TTR (**d**). Data sets were analyzed by Tukey-Kramer multiple comparison test. * *p* < 0.05; ** *p* < 0.01; *** *p* < 0.001 as compared with control (respective TTR treated with DMSO in each experiment); All experiments were performed in triplicate (means ± SEM, *n* = 3).

The dried leaves and immature fruits of *Juglans mandshurica* var. *sachalinensis* and *J. mandshurica* var. *cordiformis* (5 g each) were extracted separately with 70% methanol and 50% acetone, as shown in scheme Figure 2a. Thereafter, TTR amyloid fibril disrupting activity was evaluated by a V30M TTR Thioflavin-T (ThT) assay, shown in Figure 2b and WT TTR ThT assay, shown in Figure 2c. Also, the profound inhibitory effect of plant extract was confirmed by PROTEOSTAT^®^ Protein aggregation assay using V30M TTR peptide, as shown in Figure 2d. From these data, we found that 50% acetone extracts showed better activity than that of 70% methanol extracts for all samples.

In the ThT assay, 50% acetone extracts of immature fruits showed relatively stronger activity than that of the leaves. Further, in the PROTEOSTAT^®^ Protein aggregation assay, 50% acetone extracts of leaves of *Juglans mandshurica* var. *sachalinensis* (J1LA) showed the most potent activity, followed by 50% acetone extract of *J. mandshurica* var. *cordiformis* (J2FA) (Figure 2d). Based on these results, 50% acetone extract of immature fruits of *J. mandshurica* var. *cordiformis* was selected for further study.

### 2.2. Extraction, Fractionation and Compound Isolation of Immature Fruits of J. mandshurica var. cordiformis and Their Activities

**Figure 3:**
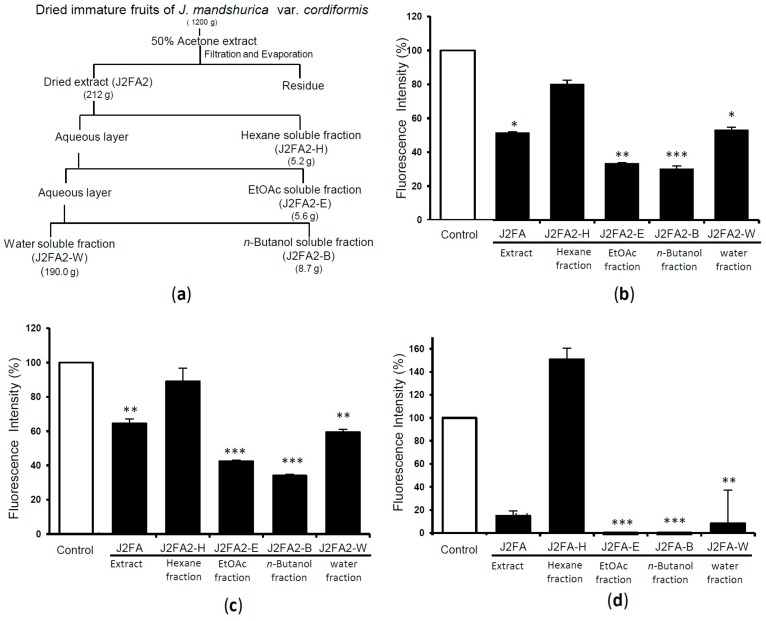
Scheme representing liquid-liquid extraction procedure and extraction yield (**a**); The amyloid fibril disrupting capacity of extract and different fractions (0.5 mg/mL) of immature fruit extract of *J. mandshurica* plants, when incubated with V30M-TTR tetramer (**b**) and WT-TTR tetramer (**c**) for 24 h, then quantified with ThT assay. Quantification was performed by with PROTEOSTAT^®^ Protein aggregation assay following similar procedures using V30M-TTR (**d**). Data sets were analyzed by Tukey-Kramer multiple comparison test. * *p* < 0.05; ** *p* < 0.01; *** *p* < 0.001 as compared with control (respective TTR treated with DMSO in each experiment); All experiments were performed in triplicate (means ± SEM, *n* = 3).

The dried immature fruits of *J. mandshurica* var. *cordiformis* (1200 g) were extracted with 50% acetone to obtain 212.0 g of dried extract (Figure 3a). The extract (J2FA2) was then suspended in water and partitioned with hexane, ethyl acetate, *n*-butanol to obtain hexane soluble (J2FA2-H), ethyl acetate (EtOAc) soluble (J2FA2-E), *n*-butanol soluble (J2FA2-B), and water soluble fractions (J2FA2-W) (Figure 3a). Each fraction was then screened for amyloid fibril disrupting activity. Results from ThT assay using V30M-TTR aggregate are shown in Figure 3b and that using WT-TTR aggregate are shown in Figure 3c. Results of the PROTEOSTAT^®^ Protein aggregation assay are shown in Figure 3d. From these data, we observed that the ethyl acetate and *n*-butanol fractions showed significant amyloid fibril disrupting activities in both ThT and PROTEOSTAT^®^ Protein aggregation assays. The ethyl acetate fraction was then subjected to Sephadex LH-20 column chromatography, followed by silica gel column chromatography and syringic acid was obtained as a main constituent. The structure of syringic acid was determined on the basis of ^1^H- and ^13^C-NMR data, comparison with literature values [24] and co-TLC with an authentic sample. Syringic acid has been previously reported as main constituents of fruits of different cultivars of *Juglans regia* [25]. The *n*-butanol fraction also afforded syringic acid as main constituent following similar purification process. However, syringic acid was inactive in amyloid fibril disrupting activity assays. As isolation and identification of other compounds was not possible at present, we preceded further with the activity evaluation of previously isolated/reported compounds from *Juglans* plants.

### 2.3. Evaluation of TTR Amyloid Fibril Disrupting Activity of Previously Isolated Compounds from Juglans Plants

**Figure 4:**
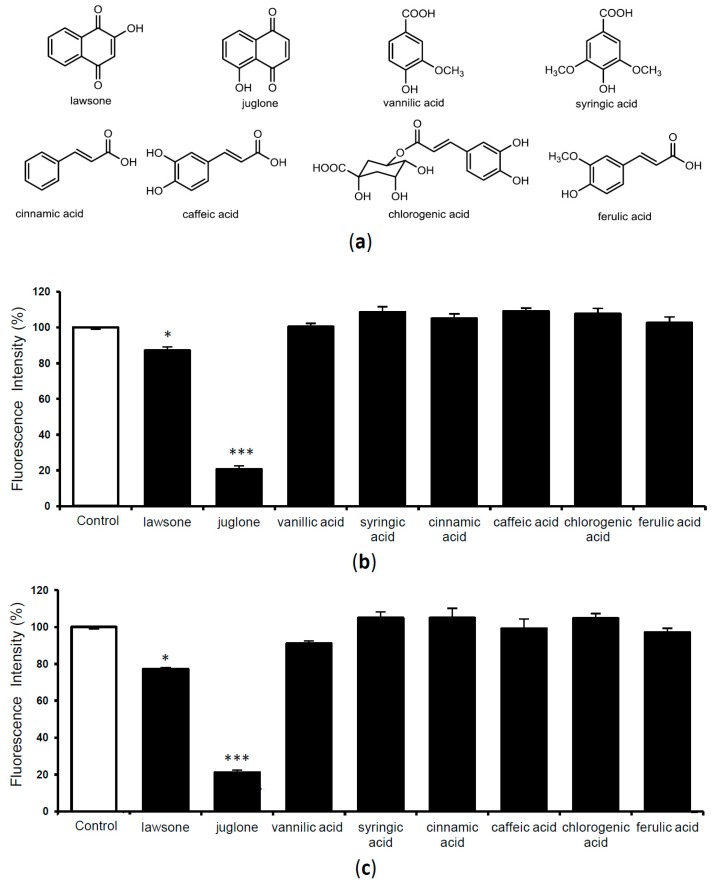
The chemical structures of compounds selected for activity evaluation (**a**). The amyloid fibril disrupting capacity of compounds (10 μM) when incubated with V30M-TTR tetramer (**b**) and WT-TTR tetramer (**c**) for 24 h, then quantified with ThT assay. Data sets were analyzed by Tukey-Kramer multiple comparison test. * *p* < 0.05; ** *p* < 0.01; *** *p* < 0.001 as compared with control (respective TTR treated with DMSO in each experiment); All experiments were performed in triplicate (means ± SEM, *n* = 3).

Naphthoquinone derivatives and phenolic compounds including benzoic acid derivatives and cinnamic acid derivatives are the reported main compounds from *Juglans* plants [15,19,25,26]. To identify the possible compounds responsible for these activities, we selected two naphthoquinone derivatives (lawsone, juglone), two benzoic acid derivatives (vanillic acid, syringic acid) and four cinnamic acid derivatives (cinnamic acid, caffeic acid, chlorogenic acid, ferulic acid) (Figure 4a) that are commercially available or previously isolated from plants to evaluate the TTR amyloid fibril disrupting activity. Activities of these compounds from the experiments after using V30M-TTR and WT-TTR aggregates are given in Figure 4b,c, respectively. Juglone (5-hydroxy-1,4-naphthoquinone), a naphthoquinone derivative showed most potent amyloid fibril disrupting activity in both assays. Another, naphthoquinone derivative, lawsone (2-hydroxy-1-4-naphthoquinone) showed weak but significant activity. All other compounds including benzoic acid derivatives and cinnamic acid derivatives did not show any activity. These results suggested that naphthoquinone derivatives might be potential sources for the development of TTR amyloid fibril disrupting agents. However, co-TLC with authentic sample of juglone showed that neither the extract nor the fractions contained juglone in our experimental samples. 

### 2.4. Dose Dependent TTR Amyloid Fibril Disrupting Activity and Cytotoxicity Evaluation of Juglone

**Figure 5:**
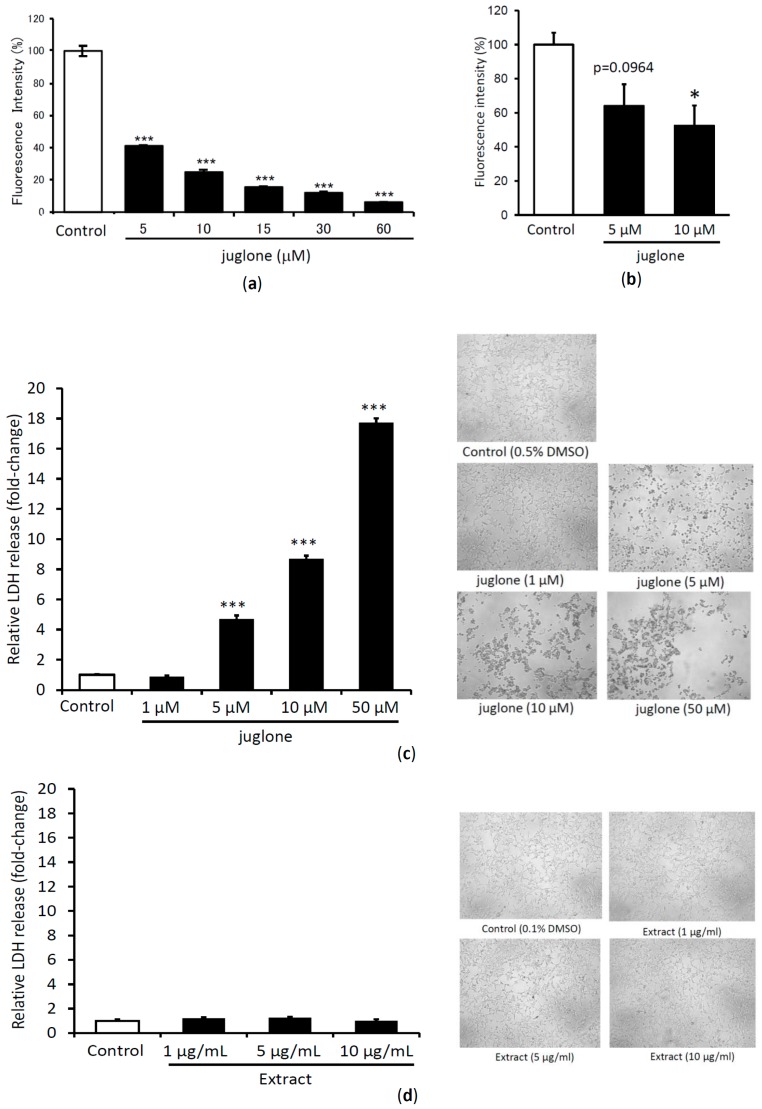
The amyloid fibril disrupting activity of different concentration of juglone when incubated with V30M-TTR tetramer for 24 h and then quantified with Thioflavin T assay (**a**) and PROTEOSTAT^®^ Protein aggregation assay (**b**). LDH release (left panel) and images of HEK293 cells treated with the different concentration of juglone (**c**) and 50% acetone extract of immature fruits of *J. mandshurica* var. *cordiformis* (**d**). Data sets were analyzed by Tukey-Kramer multiple comparison test. * *p* < 0.05; ** *p* < 0.01; *** *p* < 0.001 as compared with control (TTR treated with DMSO in each experiment for (**a**,**b**), and DMSO for (**c**,**d**)); All experiments were performed in triplicate (means ± SEM, *n* = 3).

The results above indicate that juglone has potent TTR amyloid fibril disrupting activity. The dose-dependent activity of juglone was further evaluated at different concentration range (5, 10, 15, 30 and 60 μM), and its activity was proportional to the increased concentration as assayed by ThT assay using V30M-TTR aggregates (Figure 5a). In the PROTEOSTAT^®^ Protein aggregation assay, juglone at lower concentration (5 μM) also showed amyloid fibril disrupting activity (Figure 5b).

The cytotoxicity of juglone was evaluated using HEK293 cells. Cytotoxicity was quantified using Cytotoxicity Detection Kit Plus (LDH) and images were captured using JuLI TM Br Live Cell Movie Analyzer (Figure 5c). Juglone at a concentration of 5 μM or higher showed significant cytotoxicity. Similarly, cytotoxicity of 50% acetone extract of *J. mandshurica* var. *cordiformis* was also evaluated using similar procedures, where the extract at higher concentration (10 μg/mL) also did not show any cytotoxicity.

## 3. Discussion

In physiological condition, TTR protein is present in the cerebrospinal fluid and circulation in functional soluble state [27]. However, in TTR amyloidosis, TTR gets fibrillized and deposited in organs forming the amyloid plaques which may trigger an organ dysfunction like PNS amyloidosis, CNS amyloidosis, cardiomyopathy, ophthalmopathy and nephropathy [28]. The fibrillization of TTR is considered the main event in sporadic amyloid disease, senile systemic amyloidosis (SSA), which leads to cellular toxicity on deposited sites, and results in malfunction of affected organs and sensorimotor disturbances [5,29,30]. The mechanisms responsible for the fibrillization of TTR in ATTRm amyloidosis are the consequence of one of over 100 TTR mutations with several variants that often exhibit tissue-selective deposition and pathology [28]. Familial TTR amyloidosis was accepted to be a rare endemic disease mainly distributed in Portugal, Sweden, and Japan. However, recent improvements in diagnosis revealed the wide array of patients distributed globally especially Val30Met mutant TTR. Unfortunately, there are only limited treatment options available at present. Therefore, identification of compounds that exhibit TTR fertilization inhibitory or TTR amyloid fibril disrupting activities might be potential candidates for the development of therapeutic agents in TTR amyloidosis. Natural products of plant origin can be one of the sources for lead discovery.

When TTR was recognized as T4-binding prealbumin, the main research strategy was to search for compounds that can competitively bind to TTR verses isotopic labeled T4 in the competitive binding assay. Reported natural products were salicylic acid [31], phloretin [32] and retinoic acid [33]. Following the first crystallographic TTR analysis [34] and discovery of first T4 stabilizer [35], the research strategy was changed and tended towards the search of potent TTR stabilizers that will bind effectively to the T4-binding site of TTR thus stabilizing the TTR tetramer. The most predominant reported compounds were xanthonoids from *Calophyllum teysmannii* var. *inophylloide* [36], genistein from soybeans [4] and curcumin from turmeric [37] among others. Similarly, some natural products were also reported as amyloid fibril disrupters or amyloid breakers such as doxycycline, psoromic acid, usnic acid and gossypol [38,39]. 

In the present study, we report the TTR amyloid fibril disrupting activities of extracts of leaves and immature fruits of *Juglans mandshurica* var. *sachalinensis* and *Juglans mandshurica* var. *cordiformis* using ThT and PROTEOSTAT^®^ Protein aggregation assay methods. The results suggested that the 50% acetone extracts of immature fruits were able to disrupt TTR amyloid aggregates comparatively better than other samples. Hence, 50% acetone extract of immature fruits of *Juglans mandshurica* var. *cordiformis* was selected for further fractionation. The extract was then suspended in water and extracted successively with hexane, ethyl acetate and *n*-butanol to obtain hexane, ethyl acetate, *n*-butanol and water soluble fractions. The ethyl acetate and *n*-butanol fractions showed significant activity in both assays, and both of these fractions were further subjected to column chromatography to isolate syringic acid as a main compound. However, the isolated compound syringic acid did not show any activity. As the isolation of other compounds was not successful, we selected some natural products previously reported from *Juglans* plants for further assessment. These compounds were obtained from commercial sources or previously isolated from other plants. Among these compounds, juglone showed the most potent activity in a dose-dependent manner. However, we could not confirm the presence of juglone in the extracts or fractions used in this study based on preliminary chromatographic studies including co-TLC with authentic samples. Various parts of *Juglans* plants contain hydrojulone and its glucosides, which are later converted to juglone, a main allelopathic compound, after reacting with air, enzymes and other compounds [40,41,42]. Another naphthoquinone derivative, lawsone showed weak activity. The only difference in the structure of juglone and lawsone is the attachment of hydroxyl group in the 5- and 2-positions, respectively. Juglone also showed strong cytotoxicity in HEK293 cells. Previously, various studies have been performed on juglone regarding its cytotoxicity against various cell lines [43,44,45,46,47]. Thus, future studies should focus on the isolation and identification of other naphthoquinone derivatives with potent activity and low cytotoxicity. Similarly, naphthoquinone derivatives from synthetic sources should also be evaluated to understand their structure activity relationships. Further bioactivity guided isolation is necessary to isolate and identify the active compounds from these extracts. Similarly, the possible synergistic activity of naphthoquinones and phenolic compounds should also be evaluated in future. 

In conclusion, promising TTR amyloid fibril disrupting activities of extracts of *Juglans* plants and juglone, a naphthoquinone derivative were observed. Further studies should focus on the detailed mechanism based in vitro and in vivo studies to provide sufficient scientific evidences for therapeutic uses of these samples.

## 4. Materials and Methods

### 4.1. Chemicals

Lawsone and caffeic acid were purchased from Tokyo Chemical Industry Co. Ltd. (Tokyo, Japan). Juglone and syringic acid were purchased from Sigma (St. Louis, MO, USA). Vanillic acid was purchased from Kanto Chemical Co., Inc. (Tokyo, Japan). *Trans*-cinnamic acid was purchased from Nacalai Tesque, Inc. (Kyoto, Japan). Chlorogenic acid and ferulic acid were isolated in previous studies from *Heynea trijuga* [48] and *Ligusticopsis wallichiana* [49], respectively. Thioflavin T was purchased from Wako Pure Chemical Industry Co. Ltd. (Osaka, Japan), PROTEOSTAT^®^ Protein aggregation assay was purchased from Enzo Life Sciences, Inc. (New York, NY, USA) and the Cytotoxicity Detection Kit Plus (LDH) was purchased from Roche Diagnostic GmbH (Mannheim, Germany). 

### 4.2. Plant Materials

The immature fruits and leaves of *Juglans mandshurica* Maxim. var. *sachalinensis* (Komatsu) Kitam. and *Juglans mandshurica* Maxim. var. *cordiformis* (Makino) Kitam. were collected from Medicinal Plant Garden, School of Pharmacy, Kumamoto University in May 2018 and identified by one of the authors (M.W.).

### 4.3. Extraction, Fractionation and Isolation

For screening of TTR amyloid fibril disrupting activity, 5 g of dried leaves and fruits of *J. mandshurica* var. *sachalinensis* and *J. mandshurica* var. *cordiformis* were extracted with 70% methanol and 50% acetone, separately. These extracts were then dried under reduced pressure. These extracts were then dissolved in DMSO for further analysis. For the large-scale extraction, 1200 g of dried immature fruit of *J. mandshurica* var. *cordiformis* were extracted with 50% acetone. The extract was then concentrated under reduced pressure using a rotary evaporator to obtain 212 g of extract (J2FA2). The extract was then suspended in water and partitioned successively with hexane, *n*-butanol and ethyl acetate to give hexane- (J2FA2-H), ethyl acetate- (J2FA2-E), *n*-butanol- (J2FA2-B) and water-soluble (J2FA2-W) fractions. J2FA2-E (5.0 g) was subjected to Sephadex LH-20 (Amersham Pharmacia Biotech, Tokyo, Japan) and eluted with methanol to obtain total 7 fractions based on their TLC patterns. Among them fraction 2 (3.5 g) was subjected to silica gel column chromatography (0.040–0.063 mm, Merck KGaA, Darmstadt, Germany) and eluted with CH_2_Cl_2_:MeOH:water (9:1:0.1) to obtain syringic acid (180 mg). Structure was elucidated on the basis of ^1^H- and ^13^C-NMR data (AVANCE-I 600 NMR spectrometer, Bruker, Billerica, MA, USA). ^1^H-NMR (600 MHz, CD_3_OD) δ_H_: 7.22 (2H, *s*, H-2,6), 3.78 (6H, *s*, 3-OCH_3_, 5-OCH_3_); ^13^C-NMR (150 MHz, CD_3_OD) δ_C_: 170.1 (C-7), 148.8 (C-3,5), 141.7 (C-4), 122.1 C-1), 108.3 (C-2,6), 56.8 (OCH_3_ x2). By following similar chromatographic procedures, syringic acid (30 mg) was also isolated from the *n*-butanol fraction. 

### 4.4. Thioflavin T Binding Assay

Recombinant wild-type (WT) and V30M TTRs (0.2 mg/mL, final concentration) were generated by previously described method [50,51]. The aging of amyloid fibril was completed by incubating the tetramers in 200 mM acetate buffer containing 1 mM EDTA and 100 mM KCl for 72 h (pH 3.8 or 4.4). Thereafter, aged amyloid fibrils were co-incubated with indicated concentration of plant extract for additional 24 h. and evaluated by ThT assay as previously reported method [50,51]. Fluorescence emission spectra were obtained with excitation (450 nm) and emission wavelengths (482 nm), using a FP-8500 spectrofluorometer (JASCO, Tokyo, Japan).

### 4.5. PROTEOSTAT^®^ Protein Aggregation Assay

Recombinant V30M TTR peptide (0.2 mg/mL, final concentration) was generated by previously described method [50,51]. The aging of amyloid fibril was completed by incubating the tetramers in 200 mM acetate buffer containing 1 mM EDTA and 100 mM KCl for 72 h (pH 3.8 or 4.4). Thereafter, aged amyloid fibrils were co-incubated with indicated concentration of plant extract for additional 24 h. and evaluated by PROTEOSTAT^®^ Protein aggregation assay according to the manufacturer’s protocol (Enzo Life Sciences). Fluorescence emission spectra were obtained with excitation setting (545 nm) and emission wavelength (595 nm), using an Infinite^®^ M1000 microplate reader (TECAN, Salzburg, Austria).

### 4.6. Cell Culture

The HEK293 cells were cultured in Dulbecco’s Modified Eagle’s medium (DMEM) supplemented with 10% fetal bovine serum, 100 U/mL of penicillin and 100 U/mL of streptomycin. The cells were maintained in humidified incubator at 37 °C with 5% CO_2_. Cells were sub-cultured regularly using EDTA. 

### 4.7. Cell Cytotoxicity Assay Using Cytotoxicity Detection Kit Plus (LDH) 

HEK293 cells were seeded at a density of 1.8 × 10^5^ cells/well on 24-well plate. After 48 h incubation, cells were checked for 70–80% confluence then the preceding medium is replaced by phenol red-free DMEM low glucose medium supplemented with 1% fetal bovine serum, 100 U/mL of penicillin/streptomycin and 2mM l-glutamine. 1% DMSO (control) or plant extracts/compounds at indicated concentrations were applied then incubated for 24 h. The LDH release from cells after plant extract treatment was determined with a commercial kit according to the manufacturers’ protocol (Roche). The percentage of LDH release was calculated. 

### 4.8. Statistical Analysis

For quantitative analysis, the result represents the mean ± SEM (n = 3) and the data were analyzed using one-way ANOVA with either with Tukey-Kramer multiple comparison test or (JMP software, SAS Institute, Cary, NC, USA) as indicted in each figure legend. 

## References

[1] Da Costa G., Ribeiro-Silva C., Ribeiro R., Gilberto S., Gomes R.A., Ferreira A., Mateus É., Barroso E., Coelho A.V., Freire A.P. (2015). Transthyretin amyloidosis: Chaperone concentration changes and increased proteolysis in the pathway to disease. PLoS ONE.

[2] Quintas A., Vaz D.C., Cardoso I., Saraiva M.J.M., Brito R.M.M. (2001). Tetramer Dissociation and Monomer Partial Unfolding Precedes Protofibril Formation in Amyloidogenic Transthyretin Variants. J. Biol. Chem..

[3] Chan G.G., Koch C.M., Connors L.H. (2017). Blood Proteomic Profiling in Inherited (ATTRm) and Acquired (ATTRwt) Forms of Transthyretin-Associated Cardiac Amyloidosis. J. Proteome Res..

[4] Green N.S., Foss T.R., Kelly J.W. (2005). Genistein, a natural product from soy, is a potent inhibitor of transthyretin amyloidosis. Proc. Natl. Acad. Sci. USA.

[5] Ando Y., Coelho T., Berk J.L., Cruz M.W., Ericzon B.G., Ikeda S.I., Lewis W.D., Obici L., Planté-Bordeneuve V., Rapezzi C. (2013). Guideline of transthyretin-related hereditary amyloidosis for clinicians. Orphanet J. Rare Dis..

[6] Sekijima Y. (2014). Recent progress in the understanding and treatment of transthyretin amyloidosis. J. Clin. Pharm. Ther..

[7] Benson M.D., Waddington-Cruz M., Berk J.L., Polydefkis M., Dyck P.J., Wang A.K., Planté-Bordeneuve V., Barroso F.A., Merlini G., Obici L. (2018). Inotersen Treatment for Patients with Hereditary Transthyretin Amyloidosis. N. Engl. J. Med..

[8] Adams D., Gonzalez-Duarte A., O’Riordan W.D., Yang C.-C., Ueda M., Kristen A.V., Tournev I., Schmidt H.H., Coelho T., Berk J.L. (2018). Patisiran, an RNAi Therapeutic, for Hereditary Transthyretin Amyloidosis. N. Engl. J. Med..

[9] Coelho T., Maia L.F., Da Silva A.M., Cruz M.W., Planté-Bordeneuve V., Suhr O.B., Conceiçao I., Schmidt H.H.J., Trigo P., Kelly J.W. (2013). Long-term effects of tafamidis for the treatment of transthyretin familial amyloid polyneuropathy. J. Neurol..

[10] Adams D., Buades J., Suhr O., Obici L., Coelho T. (2015). Preliminary assessment of neuropathy progression in patients with hereditary ATTR amyloidosis after orthotopic liver transplantation (OLT). Orphanet J. Rare Dis..

[11] Guo L., Giasson B.I., Glavis-Bloom A., Brewer M.D., Shorter J., Gitler A.D., Yang X. (2014). A cellular system that degrades misfolded proteins and protects against neurodegeneration. Mol. Cell.

[12] Savelieff M.G., Detoma A.S., Derrick J.S., Lim M.H. (2014). The ongoing search for small molecules to study metal-Associated amyloid-β species in alzheimers disease. Acc. Chem. Res..

[13] Korshavn K.J., Jang M., Kwak Y.J., Kochi A., Vertuani S., Bhunia A., Manfredini S., Ramamoorthy A., Lim M.H. (2015). Reactivity of Metal-Free and Metal-Associated Amyloid-β with Glycosylated Polyphenols and Their Esterified Derivatives. Sci. Rep..

[14] Okada M., Mitsuhashi H., Wada H., Terabayashi S., Kondo K., Murata J., Kikuchi G. (2002). Newly Revised Illustrated Medicinal Plants of World.

[15] Zhou Y., Yang B., Jiang Y., Liu Z., Liu Y., Wang X., Kuang H. (2015). Studies on cytotoxic activity against HepG-2 cells of naphthoquinones from green walnut husks of Juglans mandshurica maxim. Molecules.

[16] Xu H.L., Yu X.F., Qu S.C., Zhang R., Qu X.R., Chen Y.P., Ma X.Y., Sui D.Y. (2010). Anti-proliferative effect of Juglone from Juglans mandshurica Maxim on human leukemia cell HL-60 by inducing apoptosis through the mitochondria-dependent pathway. Eur. J. Pharmacol..

[17] Chauhan N., Wang K.C., Wegiel J., Malik M.N. (2004). Walnut extract inhibits the fibrillization of amyloid beta-protein, and also defibrillizes its preformed fibrils. Curr. Alzheimer Res..

[18] Muthaiyah B., Essa M.M., Lee M., Chauhan V., Kaur K., Chauhan A. (2014). Dietary supplementation of walnuts improves memory deficits and learning skills in transgenic mouse model of alzheimer’s disease. J. Alzheimer’s Dis..

[19] Guo R., Cheng Z., Huang X., Song S. (2017). Research review in the main chemical constituents and pharmacological effects of *Juglans mandshurica* Maxim. Asian J. Tradit. Med..

[20] Babula P., Adam V., Havel L., Kizek R. (2009). Noteworthy Secondary Metabolites Naphthoquinones—Their Occurrence, Pharmacological Properties and Analysis. Curr. Pharm. Anal..

[21] Fang L., Ren D., Cui L., Liu C., Wang J., Liu W., Min W., Liu J. (2018). Antifatigue, Antioxidant and Immunoregulatory Effects of Peptides Hydrolyzed from Manchurian Walnut (*Juglans mandshurica* Maxim.) on Mice. Grain Oil Sci. Technol..

[22] Selamoglu Z. (2017). Polyphenolic compounds in human health with pharmacological properties. J. Tradit. Med. Clin. Natur..

[23] Hu F.B., Stampfer M.J., Manson J.E., Rimm E.B., Colditz G.A., Rosner B.A., Speizer F.E., Hennekens C.H., Willett W.C. (1998). Frequent nut consumption and risk of coronary heart disease in women: Prospective cohort study. BMJ.

[24] Liao C.R., Kuo Y.H., Ho Y.L., Wang C.Y., Yang C.S., Lin C.W., Chang Y.S. (2014). Studies on cytotoxic constituents from the leaves of elaeagnus oldhamii maxim. In non-small cell lung cancer A549 cells. Molecules.

[25] Colaric M., Veberic R., Solar A., Hudina M., Stampar F. (2005). Phenolic acids, syringaldehyde, and juglone in fruits of different cultivars of *Juglans regia* L.. J. Agric. Food Chem..

[26] Shi A., Huang J.-W., Liu Y., Yuan K. (2013). Separation, antioxidant and antimicrobial activities of chemical constituents from exocarp of *Juglans mandshurica* Maxim. Asian J. Chem..

[27] Sekijima Y., Kelly J.W., Ikeda S. (2008). Pathogenesis of and therapeutic strategies to ameliorate the transthyretin amyloidoses. Curr. Pharm. Des..

[28] Connors L.H., Richardson A.M., Theberge R., Costello C.E. (2000). Tabulation of transthyretin (TTR) variants as of 1/1/2000. Amyloid.

[29] Westermark P., Sletten K., Johansson B., Cornwell G.G. (1990). Fibril in senile systemic amyloidosis is derived from normal transthyretin. Proc. Natl. Acad. Sci. USA.

[30] Cornwell G.G., Murdoch W.L., Kyle R.A., Westermark P., Pitkänen P. (1983). Frequency and distribution of senile cardiovascular amyloid. A clinicopathologic correlation. Am. J. Med..

[31] Ingbar S.H. (1963). Observations concerning the binding of thyroid hormones by human serum prealbumin. J. Clin. Investig..

[32] Auf’mkolk M., Koehrle J., Hesch R.D., Ingbar S.H., Cody V. (1986). Crystal structure of phlorizin and the iodothyronine deiodinase inhibitory activity of phloretin analogues. Biochem. Pharmacol..

[33] Smith T.J., Davis F.B., Deziel M.R., Davis P.J., Ramsden D.B., Schoenl M. (1994). Retinoic acid inhibition of thyroxine binding to human transthyretin. BBA Gen. Subj..

[34] Zanotti G., D’acunto M.R., Malpeli G., Folli C., Berni R. (1995). Crystal Structure of the Transthyretin–Retinoic-Acid Complex. Eur. J. Biochem..

[35] Miroy G.J., Lai Z., Lashuel H.A., Peterson S.A., Strang C., Kelly J.W. (1996). Inhibiting transthyretin amyloid fibril formation via protein stabilization. Proc. Natl. Acad. Sci. USA.

[36] Maia F., Almeida M.D.R., Gales L., Kijjoa A., Pinto M.M.M., Saraiva M.J., Damas A.M. (2005). The binding of xanthone derivatives to transthyretin. Biochem. Pharmacol..

[37] Pullakhandam R., Srinivas P.N.B.S., Nair M.K., Reddy G.B. (2009). Binding and stabilization of transthyretin by curcumin. Arch. Biochem. Biophys..

[38] Cardoso I. (2006). Doxycycline disrupts transthyretin amyloid: Evidence from studies in a FAP transgenic mice model. FASEB J..

[39] Yokoyama T., Mizuguchi M. (2018). Inhibition of the Amyloidogenesis of Transthyretin by Natural Products and Synthetic Compounds. Biol. Pharm. Bull..

[40] Ercisli S., Esitken A., Turkkal C., Orhan E. (2005). The allelopathic effects of juglone and walnut leaf extracts on yield, growth, chemical and PNE compositions of strawberry cv. Fern. Plant Soil Environ..

[41] Ercisli S., Turkkal C. (2005). Allelopathic effects of juglone and walnut leaf extracts on growth, fruit yield and plant tissue composition in strawberry cvs. “Camarosa” and “Sweet Charlie.” J. Hortic. Sci. Biotechnol..

[42] Rietveld W.J. (1983). Allelopathic effects of juglone on germination and growth of several herbaceous and woody species. J. Chem. Ecol..

[43] Kiran Aithal B., Sunil Kumar M.R., Nageshwar Rao B., Udupa N., Satish Rao B.S. (2009). Juglone, a naphthoquinone from walnut, exerts cytotoxic and genotoxic effects against cultured melanoma tumor cells. Cell Biol. Int..

[44] Inbaraj J.J., Chignell C.F. (2004). Cytotoxic Action of Juglone and Plumbagin: A Mechanistic Study Using HaCaT Keratinocytes. Chem. Res. Toxicol..

[45] Ji Y.B., Qu Z.Y., Zou X. (2011). Juglone-induced apoptosis in human gastric cancer SGC-7901 cells via the mitochondrial pathway. Exp. Toxicol. Pathol..

[46] Montenegro R.C., Araújo A.J., Molina M.T., Filho J.D.B.M., Rocha D.D., Lopéz-Montero E., Goulart M.O.F., Bento E.S., Alves A.P.N.N., Pessoa C. (2010). Cytotoxic activity of naphthoquinones with special emphasis on juglone and its 5-*O*-methyl derivative. Chem. Biol. Interact..

[47] Barathi S., Shailima Vardhini R.D., Chitra P., Indra Arulselvi P. (2013). Cytotoxic effect of juglone on human peripheral blood lymphocytes. Asian J. Pharm. Clin. Res..

[48] Devkota H.P., Joshi K.R., Malla K.J., Watanabe T., Yahara S. (2014). Phenolic Compounds from the Leaves and Twigs of Heynea trijuga. Shoyakugaku Zasshi (Jpn. J. Pharmacogn.).

[49] Devkota H.P., Adhikari B., Watanabe T., Yahara S. (2018). Nonvolatile Chemical Constituents from the Leaves of *Ligusticopsis wallichiana* (DC.) Pimenov & Kljuykov and Their Free Radical-Scavenging Activity. J. Anal. Methods Chem..

[50] Miyata M., Sato T., Mizuguchi M., Nakamura T., Ikemizu S., Nabeshima Y., Susuki S., Suwa Y., Morioka H., Ando Y. (2010). Role of the glutamic acid 54 residue in transthyretin stability and thyroxine binding. Biochemistry.

[51] Miyata M., Sato T., Kugimiya M., Sho M., Nakamura T., Ikemizu S., Chirifu M., Mizuguchi M., Nabeshima Y., Suwa Y. (2010). The crystal structure of the green tea polyphenol (−)-epigallocatechin gallate—Transthyretin complex reveals a novel binding site distinct from the thyroxine binding site. Biochemistry.

